# Patient and case characteristics associated with ‘no paramedic treatment’ for low-acuity cases referred for emergency ambulance dispatch following a secondary telephone triage: a retrospective cohort study

**DOI:** 10.1186/s13049-018-0475-4

**Published:** 2018-01-10

**Authors:** Kathryn Eastwood, Amee Morgans, Johannes Stoelwinder, Karen Smith

**Affiliations:** 1 Department of Epidemiology and Preventive Medicine, The Alfred Centre, Monash University, 99 Commercial Road, Melbourne, VIC 3004 Australia; 2 Ambulance Victoria, Victoria, Australia; 3 Emergency Services Telecommunications Authority, Victoria, Australia; 4 Department of Community Emergency Health and Paramedic Practice, Monash University, Melbourne, VIC Australia

**Keywords:** Emergency medical services, Triage., Telephone., Emergency medical service communication systems., Ambulance., Health resources.

## Abstract

**Background:**

Predicting case types that are unlikely to be treated by paramedics can aid in managing demand for emergency ambulances by identifying cases suitable for alternative management pathways. The aim of this study was to identify the patient characteristics and triage outcomes associated with ‘no paramedic treatment’ for cases referred for emergency ambulance dispatch following secondary telephone triage.

**Methods:**

A retrospective cohort analysis was conducted of cases referred for emergency ambulance dispatch following secondary telephone triage between September 2009 and June 2012. Multivariable logistic regression modelling was used to identify explanatory variables associated with ‘no paramedic treatment’.

**Results:**

There were 19,041 cases eligible for inclusion in this study over almost three years, of which 8510 (44.7%) were not treated after being sent an emergency ambulance following secondary triage. Age, time of day, pain, triage guideline group, and comorbidities were associated with ‘no paramedic treatment’. In particular, cases 0–4 years of age or those with psychiatric conditions were significantly less likely to be treated by paramedics, and increasing pain resulted in higher rates of paramedic treatment.

**Conclusions:**

This study highlights that case characteristics can be used to identify particular case types that may benefit from care pathways other than emergency ambulance dispatch. This process is also useful to identify gaps in the alternative care pathways currently available. These findings offer the opportunity to optimise secondary telephone triage services to support their strategic purpose of minimising unnecessary emergency ambulance demand and to match the right case with the right care pathway.

## Background

The increasing demand for emergency ambulances, particularly from patients not requiring specific paramedic interventions, has been reported in numerous studies [1–9]. This demand places significant strain upon costly and finite emergency ambulance resources [2, 8, 10, 11], and risks the lives and health of patients who need these skills by contributing to resource scarcity [2, 12–14]. In order to manage increasing demand some ambulance services have implemented secondary telephone triage services, whereby specific low-acuity cases undergo a nurse, doctor or paramedic-led telephone triage prior to or concurrent with an emergency ambulance dispatch [13, 15–22]. The goal of this triage is to reduce the demand for emergency ambulances by diverting suitable cases to alternative transportation or management pathways [15, 23]. The impact upon ambulance demand has varied, with some services referring up to 71% of the cases triaged for emergency ambulance dispatch [17]. The Referral Service, a secondary telephone triage service operated by Ambulance Victoria in Victoria, Australia, referred 27.6% of its cases for emergency ambulance dispatch between 2009 and 2012 [15, 24]. Whilst these decisions may have been appropriate, 44.7% of these cases were not treated by paramedics, suggesting there is opportunity for optimisation of triage processes. Using emergency ambulance resources for cases not requiring specific paramedic treatment when other options exist is an inappropriate and costly use of resources and does not provide the most suitable care for these patients.

Primary triage tools such as the Medical Priority Dispatch System have been investigated to identify cases not likely to be treated by advanced life support (ALS) paramedics, however most have used simple measures such as frequencies [4, 5, 7, 9], likelihood ratios and predictive values [3, 6, 8], which do not allow for associations or relationships to be ascertained accurately. Furthermore, investigating primary triage tools for case types associated with ‘no paramedic treatment’ is impractical because these triage tools are designed to rapidly dispatch ambulances and therefore lack granularity in their assessment of cases. Unsurprisingly, the few studies examining triage of low-acuity patients have achieved only moderate success in identifying cases unlikely to be treated by ALS paramedics [2–8]. In summary, there is little reliable research investigating the associations between paramedic treatment, low-acuity patient characteristics and triage outcomes.

Ambulance-based secondary triage systems that utilise less time-sensitive, more granular triage tools which are applied by healthcare clinicians presents a better opportunity to investigate low-acuity cases and their association with ALS paramedic treatment. Identifying cases unlikely to be treated by paramedics that appear suitable for diversion to alternative forms of transportation or care can potentially reduce the demand for emergency ambulances and better align appropriate care pathways with patient need. The aim of this study was to identify the patient characteristics and triage outcomes associated with ‘no paramedic treatment’ for cases referred for emergency ambulance dispatch following secondary telephone triage.

## Methods

### Study design

A retrospective cohort analysis was conducted of cases referred for emergency ambulance dispatch following secondary telephone triage between September 2009 and June 2012.

### Study setting

Ambulance Victoria is the sole emergency medical service (EMS) provider in the state of Victoria, Australia. Melbourne is the capital and largest city in Victoria, and during the study timeframe Ambulance Victoria responded to 1,036,114 cases in Melbourne, which had a population of 4.25 million in 2012 [25, 26]. Ambulance Victoria uses a two-tiered medical response system to respond to calls for emergency medical assistance. ALS paramedics form the base qualification level, and mobile intensive care ambulance (MICA) paramedics comprise the upper tier, possessing further interventional skills and a broader suite of pharmacological agents to treat patients [27]. The decision to respond ALS paramedics, MICA paramedics or both is made using the Advanced Medical Priority Dispatch System (AMPDS) primary triage tool and an Ambulance Victoria formulated service allocation matrix [15].

The Referral Service, a subsidiary of Ambulance Victoria, has operated 24 h a day, 7 days a week since 2003, providing secondary telephone triage to cases identified as low-acuity following primary triage. These low-acuity cases are specific case types that Ambulance Victoria has identified previously as having low paramedic treatment and transportation rates and that rarely re-present with the ambulance service within 24 h [28–30] (Ambulance Victoria: Referral Service Review: The case for expansion, unpublished report). Cases that are identified as low-acuity following the AMPDS-led primary triage are transferred to the Referral Service instead of an immediate emergency ambulance dispatch. Cases undergo secondary telephone triage by qualified paramedics or nurses using a computer-based triage algorithm to determine the most appropriate disposition for the case. This may involve the provision of self-care advice, advice to self-present at a community-based medical or health service or hospital, the dispatch of an alternative service provider including home-visiting doctors, nurses and hospital outreach programs, or the dispatch of a non-emergency ambulance or emergency ambulance. During the study period the Referral Service triaged 103,768 low-acuity cases in metropolitan Melbourne. The Referral Service have been described in more detail elsewhere [15].

### Definitions

Local protocols have been utilised in other studies to define ALS and basic life support (BLS) levels of paramedic treatment, and the latter has also been used to define low-acuity cases [4, 6–8]. In Victoria, non-emergency ambulance officers are able to provide BLS care, including cardiac monitoring, extrication and manual handling assistance, basic first aid and utilise a small range of drugs to manage patients. Therefore in Victoria, low-acuity cases that still require transportation to an emergency department should be suitable for referral to a non-emergency ambulance following secondary telephone triage.

In this study, ‘no paramedic treatment’ was defined as being when no prehospital treatment or only BLS treatment occurred (i.e. no ALS paramedic level of treatment was required). Intravenous line insertion, when it was not subsequently used for drug or fluid resuscitation, was excluded from the ALS level of treatment, which aligns with previous studies [3, 4, 6]. Paramedic treatment therefore consisted of drug or fluid administration, airway management (including oxygen therapy through to airway adjuncts), perfusion or cardiovascular support management, and mental health management including chemical or physical restraint.

### Data sources

Referral Service records were extracted from the Referral Service database and corresponding paramedic records for the cases referred for emergency ambulance dispatch were extracted and linked. These records documented patient assessment, treatment, demographic and operational information.

### Procedures

Only cases with a secondary telephone triage record and a corresponding paramedic care record were included in the study. The explanatory variables selected were patient age, gender, income status, comorbidities, pain score, triage guideline, time of call, and call-taker qualification. These were selected based on their availability in the datasets and likely association with the outcome. The categories investigated within each of these variables are listed in Table 1. The 343 triage guidelines used to triage the cases directed to the Referral Service were consolidated into 44 triage guideline groups to improve model parameter approximations and maintain the predictive power of the logistic regression [31, 32]. The 21 guideline groups with the highest frequency of usage were utilised for this study to aid identification of relationships with the greatest impact. These guideline groups contained 80.4% of the cases eligible for inclusion in this study. Using these guideline groups ensured sufficient group populations for reliable results.

**Table 1:** Explanatory variables used in the prediction modelling

Variable name	Reference category
Age	55–59 year olds (selected because it contained the mean age for the cases included in this study)
Gender	Males
Income status	The ‘above’ category
Qualification	ALS paramedics (because they triaged the largest volume of cases)
Pain level	Mild
Time of day	Day shift
Triage guideline groups	Given the outcome variable involved the presence or absence of ALS paramedic treatment, the category with the most similar paramedic treatment rate to all Victorian emergency ambulance cases (55.5%) was chosen to compare with the triage guideline groups – the urinary symptoms group (paramedic treatment rate of 55.2%)

The median wage in local government areas (LGAs) was converted to a binary variable, consisting of LGAs with a median wage above or below that of the median metropolitan Melbourne wage (AU$54,100) in 2011–12. Similarly, the time of day variable was dichotomised to correspond to the day shift (0700–1700 h) and night shift (1700–0700 h) roster that most emergency ambulance crews worked at the time of the study. The call-taker qualification variable distinguished qualified nurses, ALS paramedics and MICA paramedics. Finally pain scores recorded on a scale of 1–10 were divided into three groups: mild pain <3, moderate pain 3–6 and severe pain 7–10. According to the local protocols, any patient in the moderate or severe pain group was expected to have been treated with analgesia unless the patient declined treatment.

The reference categories for the seven categorical variables are listed and explained in Table 1.

A systematic bias evaluation was conducted, involving comparison of age, gender and triage guideline groups for cases that had a corresponding paramedic record and those that did not (but where the triage outcome indicated an ambulance had been dispatched). The gender distribution did not differ between these groups (54.9% versus 55.1% respectively; chi-square = 0.093, df = 1, *p* = 0.760). However, these groups had different mean ages (57.6 years versus 49.8 years; t(8885.5) 21.85, *p* < 0.001) and triage guideline groups (chi-square = 411.567, df = 44, *p* < 0.001). The most common triage guideline groups for the cases with a corresponding paramedic record were abdominal pain (18.2%), back pain (9.9%) and dizziness and vertigo (6.7%). For the cases referred to an emergency ambulance without a corresponding paramedic record, the most common triage guideline groups were abdominal pain (24.2%), back pain (9.1%) and psychiatric conditions (7.8%). It should be noted that a large sample size can result in high statistical sensitivity to small distribution differences, which may be apparent in the statistical difference in the triage guideline groups. Nonetheless, this systematic bias evaluation does suggest that the missing cases may have imposed some bias.

### Statistical analysis

This study utilised descriptive statistical analysis and multivariable binary logistic regression analyses to determine whether there were associations between the explanatory variables and the paramedic treatment outcome variable. All the explanatory variables were assessed for multiple collinearity and found to have no correlation likely to affect the multivariable analysis [33]. All statistical analysis was conducted using SPSS Version 23 [34].

## Results

**Fig. 1:**
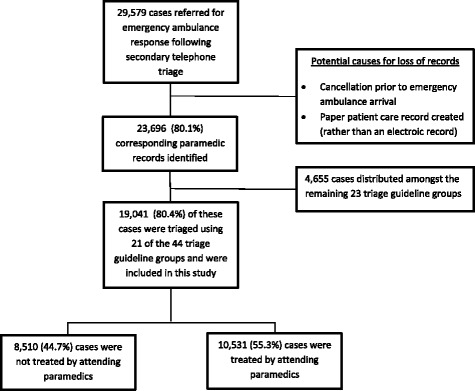
Case-flow inclusion chart

Ambulance Victoria received over one million calls to triple zero during the study timeframe and 103,768 cases had a complete Referral Service triage during this time. Of these cases, 29,579 (28.5%) were referred for an emergency ambulance dispatch. Figure 1 outlines the case selection process that resulted in 19,041 cases being eligible for inclusion.

There were 8510 cases (44.7%) were not treated by attending paramedics after having an emergency ambulance dispatched following secondary telephone triage. This ‘no paramedic treatment’ cohort of cases averaged 59.3 years of age (SD 25.3 years) and 54.4% were female.

The multivariable logistic regression model shown in Table 2 identifies that patient age, pain, triage guideline group, time of day and comorbidities variables were associated with ‘no paramedic treatment’ (*p* < 0.001). The reference categories for these variables were 55–59 year old patients (mean age), pain <3/10, urinary symptoms (which had a paramedic treatment rate most similar to all emergency ambulance cases), day shift and no comorbidities respectively. Compared to these reference categories, some of the other categories within these variables demonstrated a significant relationship with ‘no paramedic treatment’. Call-taker qualification (*p* = 0.816) and income status (*p* = 0.544) demonstrated no association with ‘no paramedic treatment’.

**Table 2:** Univariable and multivariable analyses of the explanatory variables being assessed for their association with ‘no paramedic treatment’

				Multivariable logistic regression		
Explanatory variable	Categories	Frequency	% not treated by paramedics (95% CI)	OR (95% CI)	Category *p*-value	Variable *p*-value
Gender	Male	7700	45.2 (44.2–46.3)	1	–	0.477
	Female	9256	44.2 (43.3–45.2)	1.03 (0.95–1.11)	0.477	
Age	0-4 yrs	300	92.7 (89.7–95.6)	6.14 (3.81–9.88)	<0.001	<0.001
	5-9 yrs	182	69.2 (62.5–76.0)	2.46 (1.62–3.74)	<0.001	
	10-14 yrs	202	52.0 (45.0–58.9)	1.51 (1.01–2.26)	0.047	
	15-19 yrs	436	46.3 (41.6–51.0)	1.61 (1.18–2.20)	0.003	
	20-24 yrs	656	45.6 (41.8–49.4)	1.40 (1.07–1.83)	0.015	
	25-29 yrs	847	39.3 (36.0–42.6)	1.24 (0.96–1.60)	0.098	
	30-34 yrs	1006	38.8 (35.8–41.8)	1.27 (0.99–1.61)	0.057	
	35-39 yrs	1041	34.6 (31.7–37.5)	1.10 (0.87–1.40)	0.425	
	40-44 yrs	1195	35.5 (32.8–38.2)	1.19 (0.94–1.50)	0.150	
	45-49 yrs	959	35.1 (32.1–38.2)	1.01 (0.79–1.29)	0.927	
	50-54 yrs	932	38.3 (35.2–41.4)	0.94 (0.74–1.18)	0.580	
	55-59 yrs	981	39.8 (36.7–42.8)	1	–	
	60-64 yrs	1183	40.2 (37.4–43.0)	0.97 (0.78–1.21)	0.793	
	65-69 yrs	1280	45.3 (42.6–48.0)	1.06 (0.86–1.31)	0.589	
	70-74 yrs	1519	43.8 (41.4–46.3)	0.97 (0.79–1.19)	0.758	
	75-79 yrs	1782	47.1 (44.8–49.4)	1.04 (0.85–1.26)	0.732	
	80-84 yrs	2056	50.5 (48.3–52.7)	1.18 (0.97–1.42)	0.101	
	85 + yrs	2484	52.8 (50.8–54.7)	1.16 (0.96–1.40)	0.124	
Time of day	0700-1859 h	11,621	44.6 (43.7–45.5)	1	–	<0.001
	1900-0659 h	7420	44.8 (43.7–46.0)	1.17 (1.08–1.26)	<0.001	
Income status of patients LGA	Above median income of $54 K	2499	47.6 (45.7–49.6)	1	–	0.544
	Below median income of $54 K	16,542	44.3 (43.5–45.0)	1.03 (0.93–1.15)	0.544	
Pain level	Mild (pain <3/10)	11,139	68.6 (67.7–69.4)	1	–	<0.001
	Moderate (pain 3–6/10)	2696	24.0 (22.4–25.6)	0.16 (0.14–0.18)	<0.001	
	Severe (pain >6/10)	5206	4.4 (3.8–4.9)	0.02 (0.02–0.03)	<0.001	
Triage guideline groups	Abdominal pain	4276	27.2 (25.9–28.5)	0.83 (0.63–1.08)	0.158	<0.001
	Back pain	2319	22.3 (20.5–23.9)	0.79 (0.60–1.04)	0.098	
	Dizziness	1566	58.4 (56.2–61.1)	0.77 (0.58–1.01)	0.056	
	Nausea / vomiting	1320	54.0 (51.4–56.7)	0.72 (0.55–0.95)	0.022	
	Psychiatric conditions	1087	89.8 (87.9–91.5)	4.44 (3.19–6.18)	<0.001	
	Fever	1068	62.9 (60.1–65.8)	0.86 (0.64–1.14)	0.287	
	Weakness	907	64.7 (61.6–67.8)	1.05 (0.79–1.40)	0.749	
	Headache	839	42.0 (38.6–45.3)	1.18 (0.87–1.61)	0.287	
	Confusion/Disorientation/Agitation	753	65.7 (62.3–69.0)	1.01 (0.75–1.35)	0.966	
	Flank Pain	663	14.9 (12.3–17.8)	0.68 (0.47–0.97)	0.035	
	Hip Non-Injury	526	22.2 (18.9–26.1)	0.65 (0.46–0.92)	0.016	
	Chest Pain / Discomfort	497	38.5 (34.3–42.8)	0.58 (0.42–0.81)	0.001	
	Neurological Symptoms/Transient Ischaemic Attack	436	58.3 (53.7–62.9)	0.76 (0.55–1.05)	0.097	
	Trauma	415	47.0 (42.2–51.8)	1.21 (0.85–1.72)	0.301	
	Fainting	370	61.8 (56.9–66.7)	0.79 (0.56–1.12)	0.179	
	Hypertension	370	69.4 (64.7–74.1)	1.46 (1.03–2.07)	0.033	
	Hip Injury	324	27.8 (23.0–32.7)	0.60 (0.41–0.88)	0.009	
	Diabetes-related problems	319	52.6 (47.2–58.1)	0.78 (0.55–1.11)	0.169	
	Musculoskeletal other	311	50.8 (45.2–56.4)	1.34 (0.91–1.95)	0.136	
	Diarrhoea	310	64.9 (59.5–70.2)	1.20 (0.84–1.73)	0.316	
	Urinary symptoms	365	44.8 (39.7–49.9)	1	–	
Referral Service call-taker qualification	Nurse	2700	45.3 (43.4–47.1)	1.01 (0.91–1.12)	0.903	0.816
	ALS paramedic	15,813	44.7 (43.9–45.5)	1	–	
	Intensive care paramedic	528	41.1 (36.9–45.3)	0.93 (0.74–1.17)	0.538	
Continuous variables	Mean number of comorbidities for those treated (SD)		Mean number of comorbidities for those not treated (SD)			
Comorbidities	3.15 (SD 2.3)		2.96 (2.2)	0.92 (0.91–0.94)	<0.001	<0.001

Patients under the age of 25 years were significantly more likely to receive ‘no paramedic treatment’ than 55–59 year olds (Table 2). The ‘no paramedic treatment’ rates in cases under 15 years of age ranged from 52.0–92.7% within 5-year age groups (Table 2). Cases occurring overnight were more likely to go untreated by paramedics (OR 1.17, 95% CI 1.08–1.26; *p* < 0.001) than cases that occurred during the day. With each additional comorbidity, patients became less likely to be untreated (OR 0.92, 95% CI 0.91–0.94; *p* < 0.001). Seven of the triage guideline groups demonstrated a significant association with ‘no paramedic treatment’ when compared to the reference group (Table 2). Finally, increasing pain was associated with decreasing rates of ‘no paramedic treatment’ (Table 2). Overall the multivariable analysis explained 47.3% of the variation in the outcome and the ability of the model to correctly predict the outcome was 78.6%. The sensitivity of the model was good at 87.3% and the specificity was 72.3%.

Of those categories demonstrating a statistically significant association with ‘no paramedic treatment’, the paediatric cases, psychiatric condition cases and pain cases showed the greatest association with this outcome and greatest potential for impact upon demand. The strongest association occurred for the 0–4 year old category, who were over six times more likely to be left untreated (OR 6.14, 95% CI 3.81–9.88; *p* < 0.001) than the 55–59 year old category. The mean age for cases aged 0–4 years was 1.6 years; only 7.3% of cases were treated by paramedics, and they received oxygen therapy (48.3%; *n* = 14), analgesia (24.1%; *n* = 7) and other drug administration (10.3%; *n* = 3). The most common triage guideline groups used for the 0–4 year old category were fever (38.8%, *n* = 118) and nausea and vomiting (24.7%, *n* = 75). When the 0–4 year old cases with fever were compared to the remaining 0–4 year old cases, they demonstrated paramedic treatment rates almost the same as the remaining cases (8.5% versus 9.8%; χ^2^ (1) = 0.158; *p* = 0.691). However the 0–4 year old cases with nausea and vomiting had significantly lower rates of paramedic treatment than the remaining 0–4 year old cases (1.3% versus 11.9%; χ^2^ (1) = 7.519; *p* = 0.006).

Psychiatric condition cases were over four times more likely to be untreated than the reference group (OR 4.44, 95% CI 3.19–6.18; *p* < 0.001). The mean age for this category was 44.1 years. Further investigation of the age groups in this category demonstrated no association between age and ‘no paramedic treatment’ (*p* = 0.854). The most common paramedic treatments for the 10.2% of cases treated in this category were oxygen therapy (49.5%, *n* = 55), analgesia (29.7%, *n* = 33) and mental health management (15.3%, *n* = 17). Local protocols have changed since the study period, whereby oxygen therapy is only indicated in patients with an oxygen saturation of <94%. Had this protocol been active during the study, only two psychiatric condition cases would have been indicated for oxygen therapy.

The severe pain group were 98% more likely to be treated than patients with mild pain (OR 0.02, 95% CI 0.02–0.03; *p* < 0.001), and the moderate pain group were 84% more likely to be treated than patients with mild pain (OR 0.16, 95% CI 0.14–0.18; *p* < 0.001). Mean ages for these categories were mild pain: 64.2 years, moderate pain: 56.9 years and severe pain: 51.3 years. The most common paramedic treatments for moderate and severe pain cases were analgesia (89.1%, *n* = 1864 and 98.2%, *n* = 4962 respectively) and IV access (31.3%, *n* = 654; and 47.6%, *n* = 2406 respectively). The mild pain category was most commonly treated with oxygen therapy (58.4%; *n* = 1781) and IV access (36.8%; *n* = 1122). Again, had more recent oxygen administration protocols been in place, 47.1% of the cases that received oxygen in this variable would no longer be indicated for it.

The most common triage guideline groups for patients complaining of mild pain were dizziness (13.1%; *n* = 1454), abdominal pain (11.3%; *n* = 1261) and nausea and vomiting (9.6%; *n* = 1070). The two most common triage guidelines for moderate and severe pain were abdominal pain (moderate: 33.0%; *n* = 890; severe: 40.8%; *n* = 2125) and back pain (moderate: 20.3%; *n* = 546; severe: 23.7%; *n* = 1236). Headache was the third most common triage guideline for moderate pain (5.7%; *n* = 155) and flank pain (8.4%; *n* = 435) was the third triage guideline for severe pain.

## Discussion

This is the first study to use secondary telephone triage records and paramedic records to identify factors associated with ‘no paramedic treatment’ in patients with an emergency ambulance dispatch. Five of the eight variables investigated were significantly associated with this outcome. Both the 0–4 years’ age category and psychiatric conditions triage guideline category had high rates of ‘no paramedic treatment’, whilst the pain variable demonstrated low rates of ‘no paramedic treatment’. Hence these categories appear to offer the most potential for increasing the specificity of triage decisions regarding the likely need for paramedic intervention.

Paediatric cases, particularly those under 5 years old, were over six times less likely to be treated than 55–59 year olds. This is supported by previous work showing paediatric cases had lower rates of paramedic treatment and higher rates of inappropriate usage [35–37]. The triage guideline group used to triage this age group also influenced the paramedic treatment rates, with the nausea and vomiting cases having extremely low paramedic treatment rates (1.3%), suggesting that emergency ambulance is not the most appropriate option for these cases. An investigation of whether these cases were appropriate for the ED and their temporal trends in ambulance service usage may reveal that access to alternative service providers specifically relating to childhood illness, such as specialist telephone advice lines, may allow for these cases to be managed with other care pathways [37]. Should these cases be found to be suitable for the ED, other forms of transportation to hospital such as non-emergency ambulances or transportation by the child’s family (where appropriate) should be considered.

The high rates of ‘no paramedic treatment’ for psychiatric condition cases are consistent with previous research which found these cases make up a large proportion of low-acuity work for ambulance services [12], and may be more suitable for other forms of transportation to hospital [38]. During the study period Ambulance Victoria was responsible for the transportation of psychiatric cases considered too unwell to be transported by mental health staff alone [39]. The protocol for the transportation of psychiatric cases was adjusted in 2014, after this study’s timeframe, to reflect the increasing skill level of non-emergency patient transport attendants, emergency ambulance paramedics and the increasing workload. The revised protocol now states that an emergency ambulance is to be used when the patient’s medical needs can only be met by an emergency ambulance service, and includes non-emergency ambulances as a potential option for these cases [40]. The paramedic treatment rate identified in this study supports this shift in responsibility.

Increasing pain was the most significant predictor of ‘no paramedic treatment’ (Table 2). A UK study of a secondary telephone triage services found a large proportion of pain cases were referred for emergency ambulance dispatch following triage [20]. Pain severity is not explored during the primary triage process and not considered when classifying cases as low-acuity, so the subsequent referral of severe pain cases back to emergency ambulance dispatch was not surprising. Furthermore, the strong relationship between pain and ‘no paramedic treatment’ was expected given the range of analgesic agents carried by ALS paramedics. This lack of consideration of pain severity has been a source of dissatisfaction with secondary telephone triage for patients, who feel that this process delayed access to analgesia [20]. The assessment of pain during the secondary telephone triage process could be refined to determine whether an expedient alternative response for managing pain could be introduced into the alternative care pathways. This could include a referral to a home-visiting doctor or the development of a pain pathway that allows patients with chronic pain to have a telephone consultation with a pain specialist. This may allow the provision of advice on how to use their current resources and medications to manage this acute episode whilst arranging an appointment with their regular pain management healthcare worker. For patients requiring ED assessment in Victoria, non-emergency ambulance officers have analgesia that could be used during transportation of these patients.

On initial inspection the categories and variables that had high rates of paramedic treatment suggest that these cases should have had an emergency ambulance dispatched without secondary telephone triage. However, in some instances these cases may only represent a small proportion of the total number of these case types that underwent a secondary telephone triage, and other cases within this case type may have successfully been referred away from emergency ambulance dispatch. They may constitute a sub-cohort that was successfully identified by the call-takers as requiring emergency ambulance dispatch. Therefore, further investigation is required before any recommendations can be made. Moreover, a study of the care delivered in the ED, and in the primary care setting should be conducted to direct the development of alternative care pathways and identification of cases suitable for these pathways.

Finally, the identification of ‘no paramedic treatment’ was used in this study, and in others [2–8], to suggest that cases falling into this category are not suitable for emergency ambulance attendance. However, there may be some cases that have low rates of paramedic treatment that are suitable for an emergency ambulance. There are a range of non-clinical indicators for appropriateness when considering emergency ambulance transport [41], which predominantly relate to patient welfare, safety and a responsibility of the ambulance services to ultimately ‘do the right thing’ as part of a bigger healthcare system [41]. When optimising secondary telephone triage systems, key questions should be incorporated to identify some of these situations (e.g. an unattended minor), and the ability to override the system disposition will ensure that call-takers are able to dispatch an emergency ambulance in situations where they feel it is appropriate.

### Limitations

This was a retrospective analysis using predefined variables that were not primarily devised to ascertain relationships. A potential for systematic bias was exerted by the 19.1% of cases that could not be linked to a paramedic record. The direction of this bias could not be definitively ascertained, however the lack of a paramedic record may indicate the patient was not transported by paramedics. In developing the logistic regression model, reference categories had to be chosen for comparison between the categories (the rationale for the reference categories is given in the study methodology). This makes the outcomes relative to this category and can make generalisability difficult.

Secondary telephone triage systems embedded in ambulance services differ in their operational structures, alternative service providers and staff qualifications, therefore potentially altering triage outcomes. The operation of ambulance services, and structure of primary healthcare systems within which these ambulance services operate differ between regions influencing the case-mix for secondary telephone triage. These factors may limit the generalisability of the results.

Finally, the researchers acknowledge that using paramedic treatment to identify cases suitable for alternative management streams does not take into consideration the non-clinical management skills and knowledge paramedics have, and alternative reasons why a patient might be considered suitable for an emergency ambulance dispatch. Similarly, the definition of low-acuity used in this study was limited to emergency ambulance suitability, and no ED or hospital outcomes were included in the definition.

## Conclusion

This study has highlighted that case characteristics can be used to identify particular case types that may benefit from care pathways other than emergency ambulance dispatch. This process is also useful to identify gaps in the alternative care pathways that may allow some case types to avoid entering the emergency care pathways. These findings offer the opportunity to optimise secondary telephone triage services to achieve their strategic purpose of minimising unnecessary emergency ambulance demand and match the right case with the right care pathway.

## Abbreviations

ALS: Advanced life support
AMPDS: Advanced Medical Priority Dispatch System
BLS: Basic life support
EMS: Emergency medical service
IV: Intravenous
LGA: Local government area
MICA: Mobile intensive care ambulance
